# Mdm20 Modulates Actin Remodeling through the mTORC2 Pathway via Its Effect on Rictor Expression

**DOI:** 10.1371/journal.pone.0142943

**Published:** 2015-11-23

**Authors:** Kunihiko Yasuda, Mayumi Takahashi, Nozomu Mori

**Affiliations:** The Department of Anatomy and Neurobiology, Nagasaki University School of Medicine, Nagasaki, Japan; University of Sydney, AUSTRALIA

## Abstract

NatB is an N-terminal acetyltransferase consisting of a catalytic Nat5 subunit and an auxiliary Mdm20 subunit. In yeast, NatB acetylates N-terminal methionines of proteins during *de novo* protein synthesis and also regulates actin remodeling through N-terminal acetylation of tropomyosin (Trpm), which stabilizes the actin cytoskeleton by interacting with actin. However, in mammalian cells, the biological functions of the Mdm20 and Nat5 subunits are not well understood. In the present study, we show for the first time that Mdm20-knockdown (KD), but not Nat5-KD, in HEK293 and HeLa cells suppresses not only cell growth, but also cellular motility. Although stress fibers were formed in Mdm20-KD cells, and not in control or Nat5-KD cells, the localization of Trpm did not coincide with the formation of stress fibers in Mdm20-KD cells. Notably, knockdown of Mdm20 reduced the expression of Rictor, an mTORC2 complex component, through post-translational regulation. Additionally, PKCα^S657^ phosphorylation, which regulates the organization of the actin cytoskeleton, was also reduced in Mdm20-KD cells. Our data also suggest that FoxO1 phosphorylation is regulated by the Mdm20-mTORC2-Akt pathway in response to serum starvation and insulin stimulation. Taken together, the present findings suggest that Mdm20 acts as a novel regulator of Rictor, thereby controlling mTORC2 activity, and leading to the activation of PKCα^S657^ and FoxO1.

## Introduction

The maintenance of protein homeostasis is important for anti-aging and longevity [1–3] because many fundamental protein activities, such as protein synthesis and degradation, are required for cell survival; however, various metabolic responses are reduced and suppressed with aging. By contrast, protein post-translational modifications, such as phosphorylation, acetylation, and ubiquitination, are necessary for maintaining protein homeostasis by modulating enzymatic activity, protein stabilization, and cellular localization. Thus, it can be argued that post-translational modifications are involved in regulating aging and longevity. In the case of acetyl modifications, Sirtuin is a well characterized NAD-dependent deacetylase that is linked to longevity because it increases cellular life span by activating forkhead box O (FoxO) family proteins (FoxOs) [4–6]. In addition, the mammalian target of rapamycin (mTOR) and Akt are serine/threonine kinases and also aging- and longevity-related genes that are involved in cell survival, nutrient metabolism, protein synthesis, autophagy induction and cell migration [7, 8].

Members of the N-terminal acetyltransferase (Nat) family acetylate N-terminal amino acids during *de novo* protein synthesis in eukaryotes [9–11]. Approximately 80–90% of human proteins (compared with 50–70% of yeast proteins) are acetylated at the N-terminus. However, recent studies indicated that Nat family enzymes also function as biological regulators of processes other than *de novo* protein synthesis. When in complex with Mdm20/Naa25 and Nat3/hNat5/Naa20, which are auxiliary and catalytic subunits of NatB, respectively, NatB acetylates the N-terminal methionine residues of Met-Glu, Met-Asp, and Met-Asn peptides [12, 13]. NatB also regulates actin remodeling by modulating the interaction between Tropomyosin (Trpm) and actin filaments through the N-terminal acetylation of Trpm [14–17]. Starheim et al. also reported that the reduction in the level of hNatB by siRNA knockdown (KD) inhibits cell growth and disturbs cell cycle progression in human cells [18]. Recently, we reported that the hMdm20/Naa25 complex negatively regulates poly-Q aggregate clearance by inhibiting autophagy induction through Akt phosphorylation [19]. Furthermore, Mdm20 is highly expressed in neurons, suggesting that it may be a key molecule not only in neurogenesis, but also in protein homeostasis in the brain [20].

Here, we show that Mdm20 is involved in actin remodeling and cellular motility in human cells independently of Nat5 and the Trpm-actin interaction. We also demonstrate that Mdm20 deficiency suppresses mTORC2 activity by reducing Rictor expression, suggesting a novel role for Mdm20 in modulating actin remodeling. Additionally, Mdm20 modulated pFoxO1 expression under serum starvation and insulin stimulation conditions. Taken together, these findings suggest that Mdm20 is a novel regulator of cellular homeostasis, cell motility and metabolic responses, via its ability to modulate Akt, PKCα, and FoxO1 activities in the Mdm20-mTORC2 pathway.

## Results

### 1. Cell growth suppression in Mdm20-KD cells

**Fig 1:**
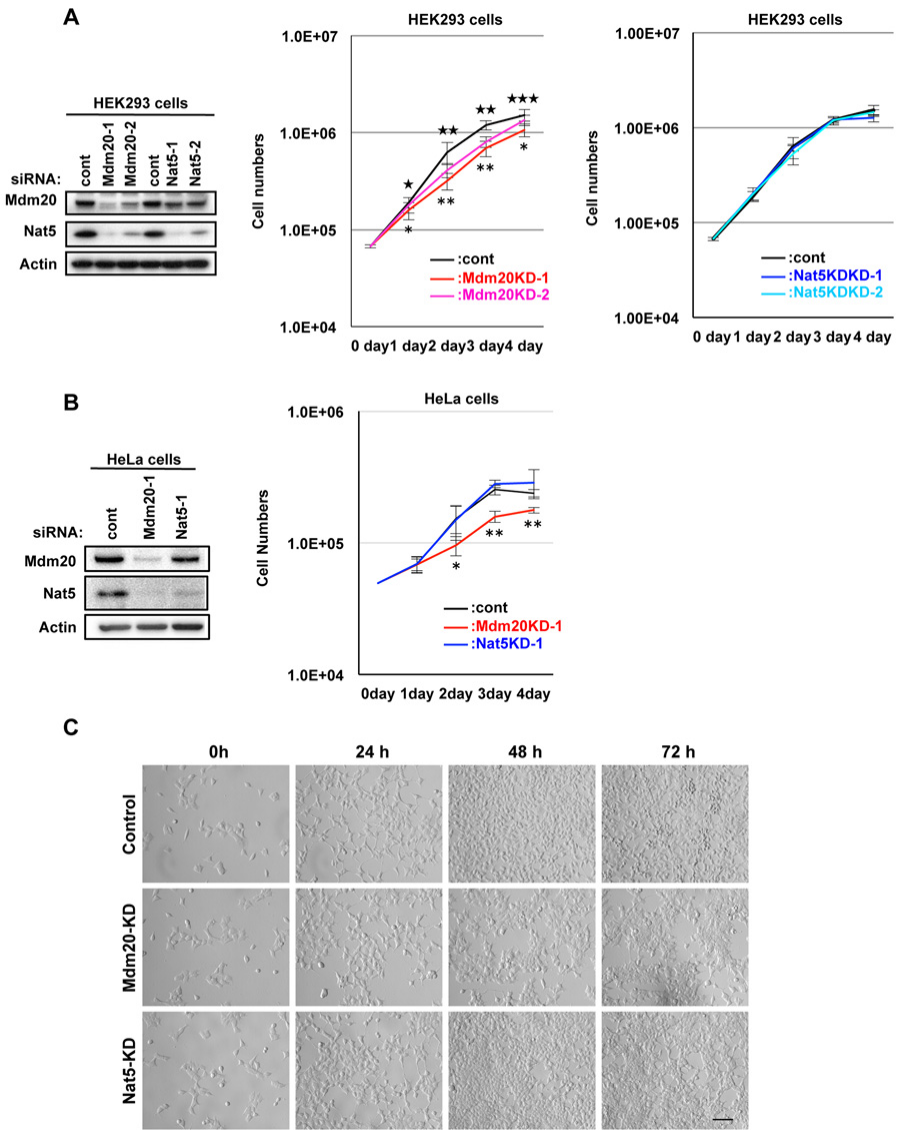
Mdm20 deficiency suppresses cell growth. A. Growth curve for Mdm20-KD and Nat5-KD HEK293 cells. The left panel shows the effectiveness of the siRNAs (control, Mdm20, and Nat5) 72 h post-transfection. After the transfection of cells with control, Mdm20, and Nat5 siRNA oligonucleotides, the number of cells was counted at the indicated time points and compared with the number of control cells. The data represent the mean ± the S.D. (n = 5). ^★^P<0.05, ^★★^P<0.005 and ^★★★^P<0.0005 indicate statistical significance between control and Mdm20-KD-1 cells. *P<0.005 and **P<0.0005 indicate statistical significance between control and Mdm20-KD-2 cells. B. Mdm20- and Nat5-KD HeLa cells displayed suppressed cell growth. Left panel: Western blot showing the effectiveness of the siRNAs (control, Mdm20 and Nat5). Right panel: Evaluation of cell growth using a similar method to A. The data represent the mean ± the S.D. (n = 5). *P<0.02 and **P<0.0001 indicate statistical significance between control and Mdm20-KD cells. C. Representative images of HEK293 cell growth at 0, 24, 48 and 72 h after siRNA transfection (Scale bar: 100 μm.).

**Fig 2:**
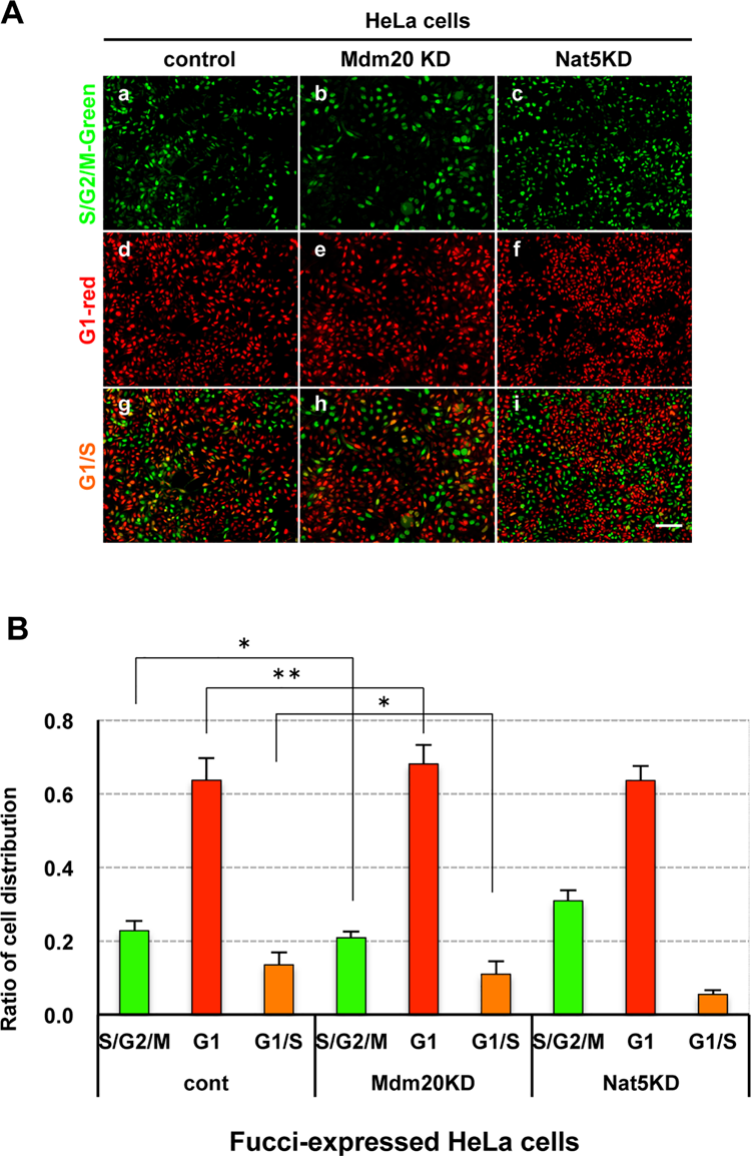
Effect of NatB deficiency on cell cycle progression. A. Cell cycle progression distribution in Fucci-expressing HeLa cells after transfection with control, Mdm20-, and Nat5 siRNA oligonucleotides. The upper panel (green: a, b, and c) and middle panel (red: d, e, and f) show S/G2/M and G1 phases, respectively. The G1/S interphase is shown as a merged image of the cells in panels g, h, and i. (Scale bar: 50 μm.) B. Comparison of cell cycle progression in Mdm20- and Nat5-KD cells. The percentage of cells in each cell cycle phase (green: S, G2 and M phase; red: G1 phase, and orange: G1/S interphase) was calculated as the total number of cells. The data represent the mean ± the S.D. (n = 3). *P<0.02 and **P<0.005.

NatB, in complex with Nat5 and Mdm20, plays a role in N-terminal acetylation during *de novo* protein synthesis in yeast. However, we found that Mdm20 deficiency, not Nat5 deficiency, suppressed the growth of human embryonic kidney 293 (HEK293) cells (Fig 1A and 1C). The cell growth suppression was predominantly observed 48 h after the transfection of HEK293 cells with two types of Mdm20 siRNA oligonucleotides (Mdm20-1 and -2), and was not detected in the first 24 h. The inhibition of cell growth coincided with the suppression of Mdm20 expression, which was detected for 48 h after transfection. The growth suppression in Mdm20-KD HEK293 cells was also confirmed in HeLa cells, a cultured cell line of human cervical cancer cells (Fig 1B). Interestingly, although the amount of Nat5 was reduced in Mdm20-KD cells, the amount of Mdm20 was only slightly reduced in Nat5-KD cells (Fig 1A and 1B). These data suggest that the growth suppression was caused by reduced Mdm20, but not Nat5 expression. Furthermore, we observed that Mdm20-KD cells were less mobile than control or Nat5-KD cells (data not shown). Starheim et al. reported that human NatB is important for cell cycle progression [18]. To examine the effect of Mdm20 on the cell cycle, we used HeLa cells stably expressing -fluorescent ubiquitination-based cell cycle indicator (Fucci) (Fig 2). Fucci-expressing cells can be used to detect cell cycle progression and division in living cells because they change in color from red in G1 phase to orange in G1/S interphase, and finally to green in the S, G2, and M phases (Fig 2A) [21]. Compared with the control cells (G1, 64%; S/G2/M, 23%; and G1/S, 13%), 68% of Mdm20-KD cells were in G1 phase, 21% were in the S/G2/M phase, and 11% were in the G1/S interphase (Fig 2B). In Nat5-KD cells, the ratio of S/G2/M phase cells was slightly increased (31%), whereas the proportion of G1/S phase cells (6%) was reduced. These data show that the distribution of the cell cycle was not substantially affected by the expression level of Mdm20, but was slightly altered by changes in Nat5 levels.

### 2. Cellular motility and configuration of actin fibers in Mdm20-KD cells

**Fig 3:**
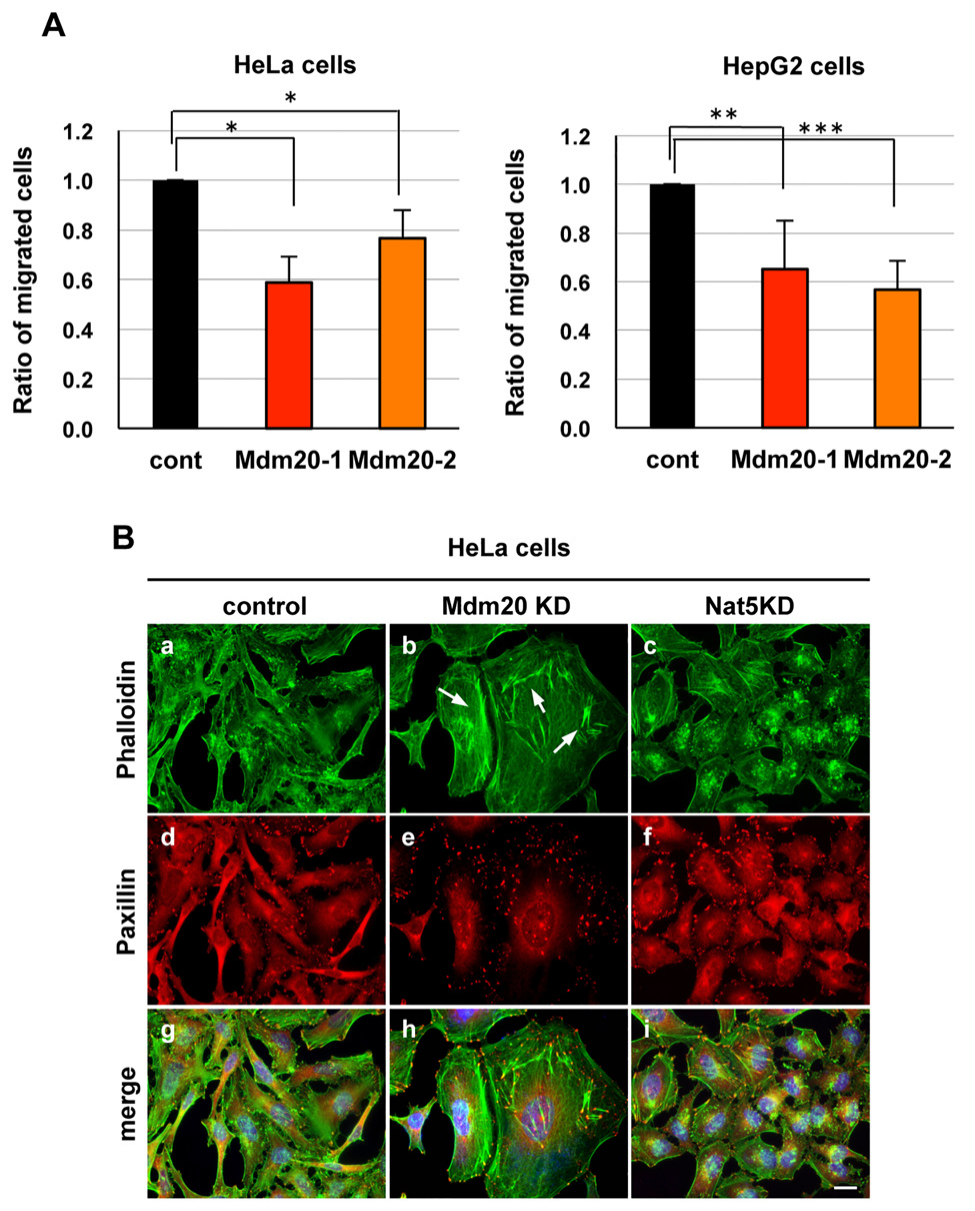
Mdm20 modulates cellular motility via its effects on the actin cytoskeleton. A. Cell migration assay using Mdm20-KD HeLa (left) and HepG2 cells (right). Seventy-two hours after transfection with two types of Mdm20 siRNA oligonucleotides, cells were applied to a Transwell chamber and incubated for 6 h. Non-migrating cells were then removed, and migrating cells were counted. The data represent the mean ± the S.D. (n = 3). *P<0.01, **P<0.005, and ***P<0.002. B. Immunohistochemistry showing the cellular localization of actin (upper panels: a-c) and paxillin (lower panels: d-f) in HeLa cells transfected with siRNA oligonucleotides. Merged images of cells counterstained with DAPI (blue) are shown in panels g, h, and i. The cells were fixed and the images were captured 72 hr post-transfection. The arrows indicate stress fibers. (Scale bar: 20 μm.)

NatB is required for actin remodeling and cellular motility through the N-terminal acetylation of Trpm. To examine this function, we performed a cell migration assay using HeLa and HepG2 cells. HeLa cell motility was lower (Mdm20-KD1, 58% and Mdm20-KD2, 75%) than that of the control cells (Fig 3A). Similarly, cellular motility was approximately 60% lower (Mdm20-KD1, 65%; and Mdm20-KD2, 57%) in HepG2 cells. As cellular motility is regulated by the assembly and disassembly of actin-formed filopodia, lamellipodia and stress fibers, we also examined the configuration of actin in Mdm20-KD cells. Interestingly, the Mdm20-KD cells were slightly larger than the control and Nat5-KD cells and fascicles of stress fibers were observed in Mdm20-KD cells, but not in Nat5-KD cells (Fig 3B-b and S1 Fig; arrow).

**Fig 4:**
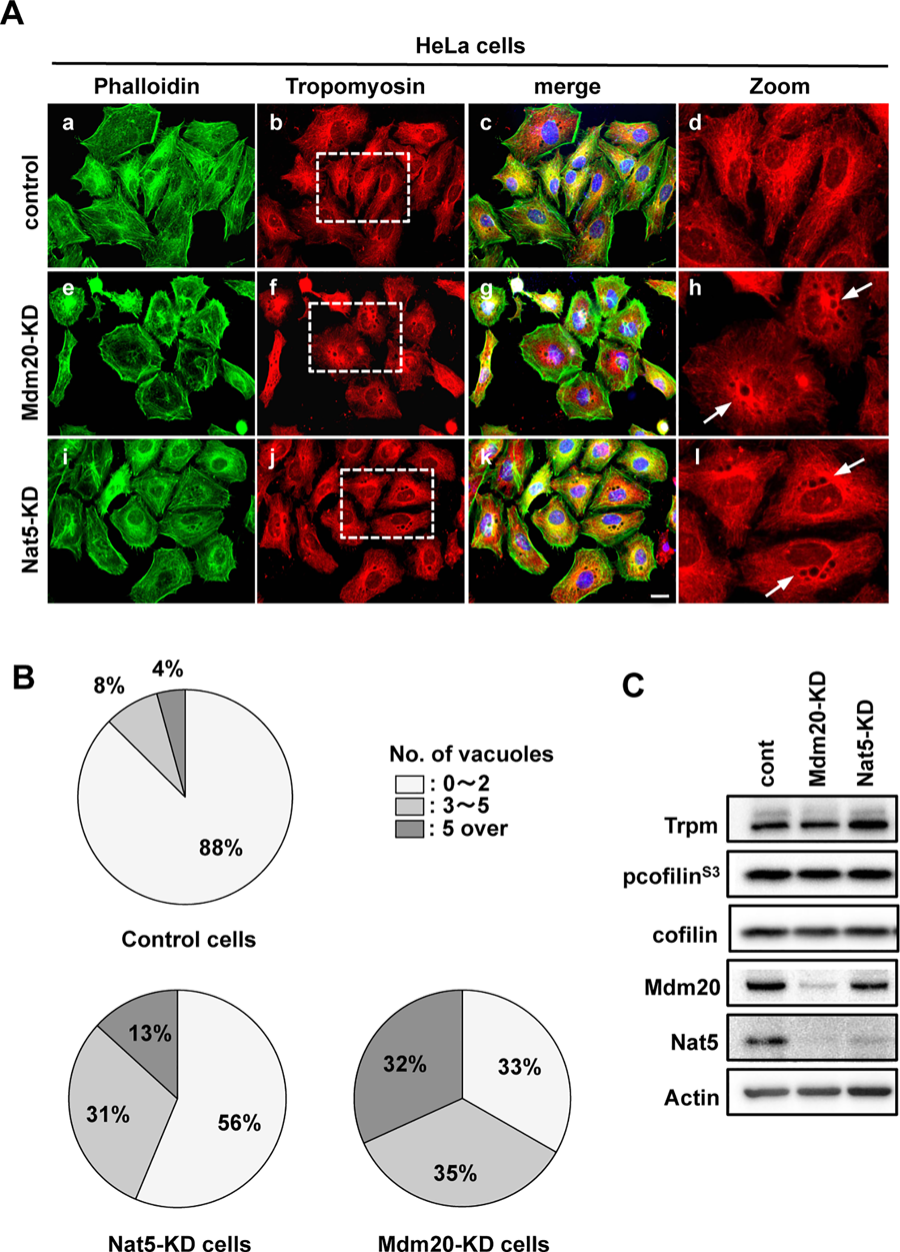
NatB affects Trpm assembly but is not linked to actin fiber formation. A. Distribution of actin and Trpm in Mdm20- and Nat5-KD HeLa cells. After fixing HeLa cells with paraformaldehyde, the cells were immunostained with phalloidin (green: a, e, and i) and anti-Trpm (red: b, f, and j) antibodies. Merged images of cells counterstained with DAPI (blue) are shown in panels c, g, and k. Low magnification (b, f, and j) and partial zoom up magnification views (d, h, and l) of the indicated regions (dotted line squares) are shown. Arrows indicate the vacuoles. (Scale bar: 20 μm.) B. Comparison of the number of vacuoles in Mdm20- and Nat5-KD cells. Cells were classified into three categories based on the number of vacuole-like structures formed, category 1 (0 to 2 (white area)), category 2 (3 to 5 (light gray area)) and category 3 (over 5 (dark gray area)). The relative ratios of the three categories were calculated based on total cell number. The data represent the mean ± the S.D. (n = 5). C. Western blots for Trpm and cofilin in HeLa cells transfected with siRNA oligonucleotides.

NatB is required for the N-acetylation of Trpm, which interacts with and stabilizes actin cables in yeast, drosophila, and mammalian cells [16, 17, 22]. Here we examined the configuration and relationship between Trpm and actin fibers to elucidate whether the conformation of stress fibers in Mdm20-KD cells is influenced by defective N-terminal acetylation of Trpm. In the control cells, Trpm fibers spread from a single origin of Trpm assembly located near the perinuclear region to throughout the cytoplasm (Fig 4A-d). By contrast, vacuoles of various sizes and numbers were observed close to the perinuclear regions in both Nat5-KD and Mdm20-KD cells (Fig 4A-h and 4A-l). In yeast, the vacuole inheritance phenotype is observed in *∆nat3* and *∆mdm20* mutants [16]. This phenotype is closely linked to actin-Trpm interaction because *act1* and *tpm1* mutants, which also display the same phenotype. Fig 4B shows the number of observed vacuoles in control and siRNA oligonucleotide-transfected cells, which were classified into three categories based on the number of vacuoles present in each cell (category 1, 0 to 2 vacuoles; category 2, 3 to 5 vacuoles; and category 3, >5 vacuoles). Although control cells predominantly belonged to category 1 (87%), only 56% and 33% of Nat5- and Mdm20-KD cells, respectively, were classified in this category. By contrast, the proportion of category 2 and 3 cells increased to 30% and 13%, respectively, among Nat5-KD cells. Furthermore, among Mdm20-KD cells, the proportion of category 3 cells increased to 31%, which was markedly higher than that found among control cells (4%). These vacuolar phenotypes were closely related with the dose-dependent expression of NatB components, as only Nat5 expression was reduced in Nat5-KD cells, whereas both Mdm20 and Nat5 expression was reduced in Mdm20-KD cells (Figs 1A and 4C). By contrast, Trpm expression levels were not affected in either Mdm20- or Nat5-KD cells (Fig 4C). These data suggest that the configuration of Trpm in Mdm20- and Nat5-KD cells is modulated by the N-terminal acetylation activity of NatB, similar to what has already been reported in yeast. However, stress fiber formation in Mdm20-KD cells did not correlate with the abnormal Trpm configuration.

Cofilin is a well-known member of the actin-depolymerization factor family and regulates the disassemblage of actin filaments [23]. LIMK or TESK phosphorylates the Ser3 of cofilin and enhances actin polymerization by inhibiting actin binding to cofilin [24, 25]. However, the phosphorylation of cofilin in Mdm20-KD cells was unchanged (Fig 4C). Cofilin was localized to the cytoplasm of Mdm20-KD cells, similar to control cells (S2 Fig). These data suggest that stress fiber formation in Mdm20-KD cells does not require the Nat5-mediated N-terminal acetylation of Trpm or enhancement of the assembly/disassembly activity of actin by cofilin.

### 3. Mdm20 regulates mTORC2 activity through Rictor expression

**Fig 5:**
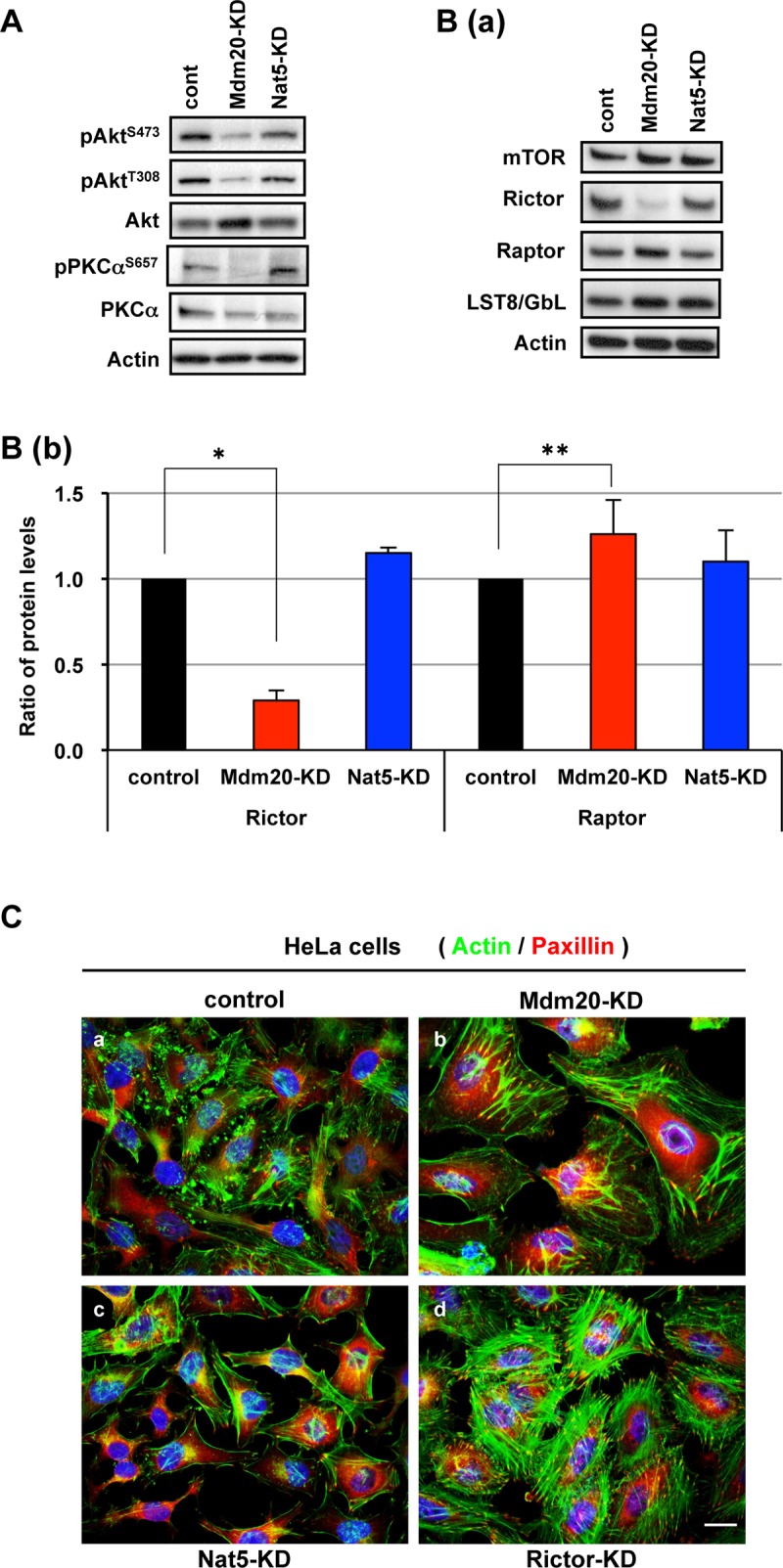
Mdm20 modulates mTORC2 activity through Rictor expression. A. Western blots for Akt and PKCα with a focus on the phosphorylated forms. HEK293 cells were transfected with control, Mdm20, and Nat5 siRNAs and after 72 h, the transfected cells were harvested and cell lysates were prepared and processed for Western blot analysis. The antibodies used are indicated. B. Western blotting was used to analyze mTORC2 activity. (a) Western blots using the indicated antibodies. (b) The amounts of Rictor and Raptor were calculated relative to those of cell extracts from control siRNA-transfected cells. The data represent the mean ± the S.D. (n = 5). *P<0.0001 and **P<0.01. C. Distribution of actin and paxillin in Mdm20-, Nat5-, and Rictor-KD HeLa cells. After fixing the HeLa cells with paraformaldehyde, the cells were immunostained with phalloidin (green) and anti-paxillin (red) antibodies and were then counterstained with DAPI (blue). (Scale bar: 20 μm.)

To reveal the mechanism of actin remodeling by Mdm20, we focused on the observation that pAkt^S473^ levels were reduced in Mdm20-KD cells. Akt is a serine/threonine protein kinase that is activated by phosphatidylinositol 3-kinase (PI3K) in response to extracellular signals, such as insulin [26]. After participating in the insulin/IGF-PI3K signaling pathway, Akt is phosphorylated at Thr308 by PDK1. However, Ser473 of Akt is also phosphorylated by mTORC2/PDK2 and this phosphorylation facilitates the phosphorylation of Akt^T308^ by PDK1 [27]. In addition to Akt, PKCα and Serum and glucocoriticoid-inducible kinase (SGK) are also substrates of mTORC2 [28–30]. PKCα is phosphorylated at Ser657 by mTORC2 and regulates actin remodeling. Here, we investigated whether Mdm20 regulates pPKCα^S657^ through mTORC2. In Mdm20-KD cells, the pPKCα^S657^ and pAkt^S473^ levels were similarly reduced (Fig 5A). Consistent with this result, the expression of Rictor, an mTORC2 component, was also significantly reduced (29%) in Mdm20-KD cells (Fig 5B). By contrast, Rictor expression was unaffected in control and Nat5-KD cells. On the other hand, the expression of Raptor, an mTORC1 component, was similar between control and Mdm20-KD cells. We confirmed that stress fiber formation is increased in Rictor-KD cells, similar to that observed in Mdm20-KD cells (Fig 5C). These data indicate that Mdm20 modulates actin cytoskeleton organization through the mTORC2-PKCα pathway.

### 4. Regulation of Rictor expression in Mdm20-KD cells

**Fig 6:**
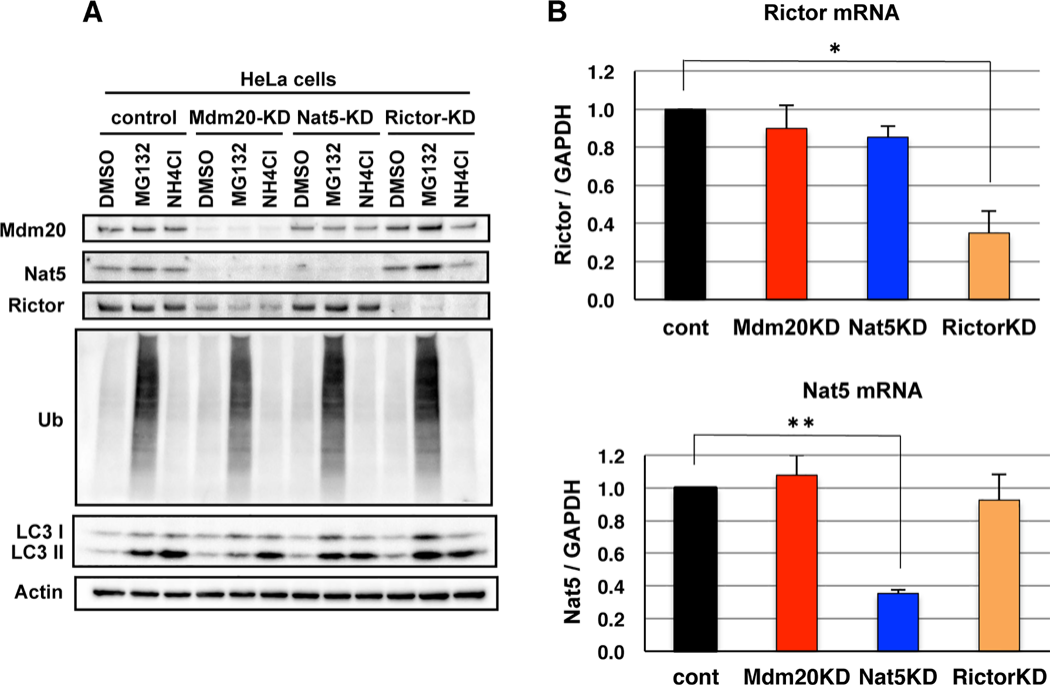
Reduction in Rictor expression in Mdm20-KD cells is related to post-translational but not transcriptional regulation. A. Western blots showing the Rictor and Nat5 protein in Mdm20-, Nat5- and Rictor-KD cells treated with MG132 (0.2 μM) or NH_4_Cl (7.5 mM). B. Quantitative real-time PCR for Nat5 and Rictor mRNA expression was performed using reverse transcription products obtained from control, Mdm20-, Nat5-, and Rictor-KD cells. GAPDH mRNA was used as an internal control for this assay. The data represent the mean ± the S.D. (n = 3). *P<0.01 and **P<0.0005.

To determine how Mdm20 regulates the expression of Rictor and Nat5, we examined the effects of MG132 (a proteasome inhibitor) and NH_4_Cl (a lysosomal protease inhibitor) on protein turnover in Mdm20-KD cells (Fig 6A). However, in the presence of either of these inhibitors, neither Rictor nor Nat5 showed increased expression in Mdm20-KD cells. These data indicate that Nat5 and Rictor expression is not regulated by the protein degradation system in Mdm20-KD cells. We also investigated the transcription of Rictor and Nat5 mRNAs in Mdm20-KD cells using quantitative PCR. Unlike protein expression, Rictor and Nat5 mRNA levels were not reduced in Mdm20-KD cells (Fig 6B). Taken together, these data suggest that Mdm20 regulates the expression of Nat5 and Rictor during translation, but not via transcriptional regulation.

### 5. FoxO1 regulation by Mdm20 through mTORC2 and Akt activation

**Fig 7:**
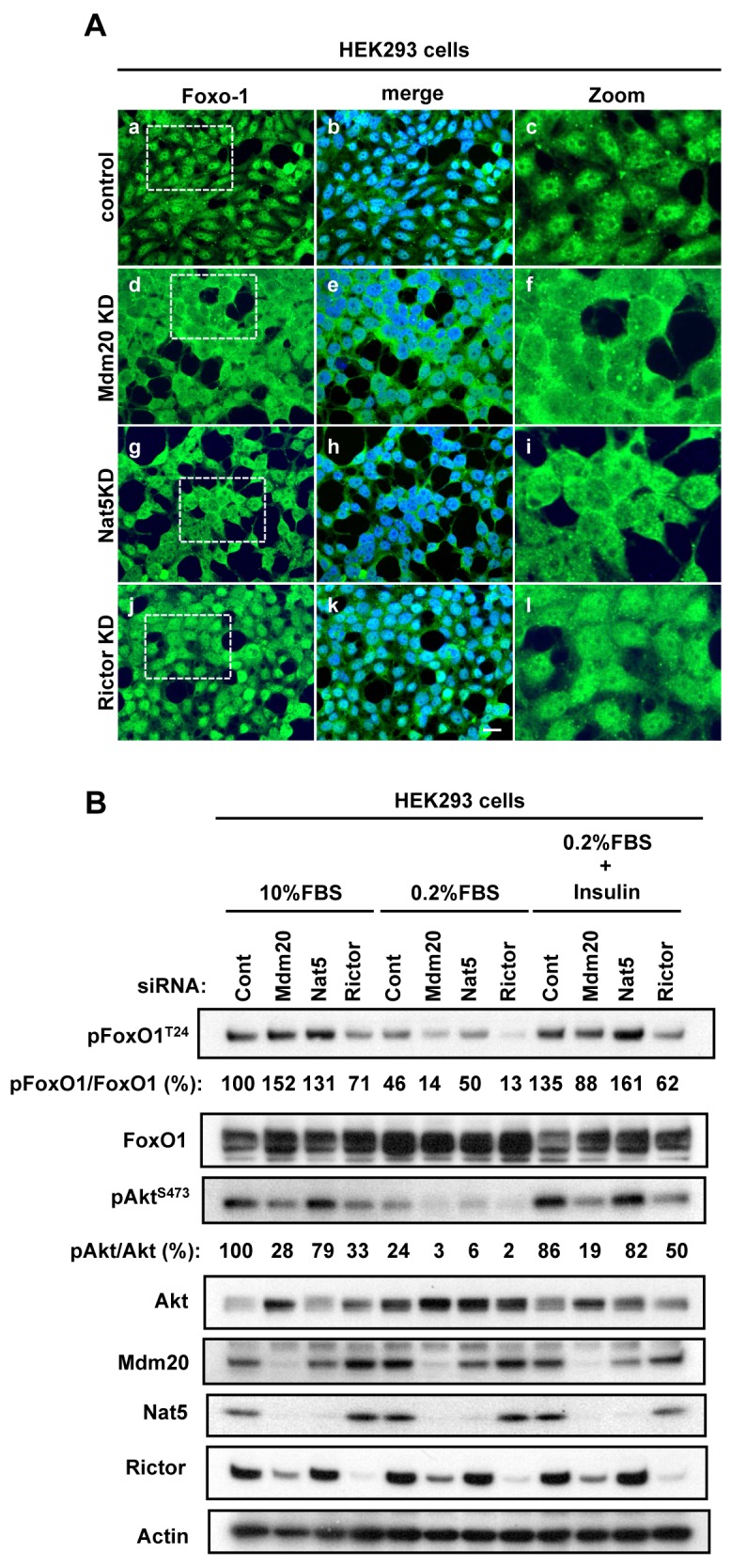
Effect of reduced levels of Mdm20 on FoxO1 localization and phosphorylation in Mdm20-KD cells. A. Immunohistochemistry against FoxO1 in Mdm20-, Nat5- and Rictor-KD HEK293 cells. Panels a, d, g and j are immunostained with anti-FoxO1 antibody; these images are merged with DAPI (blue) staining in panels b, e, h, and k. Low magnification (a, d, g, and j) and partial zoom up views (c, f, i, and l) of the indicated area (dotted line squares) are shown. (Scale bar: 20 μm.) B. Western blots of pFoxO1 to evaluate its induction in response to insulin stimulation. Control, Mdm20-KD, Nat5-KD, and Rictor-KD HEK293 cells were serum starved (0.2% FBS) for 20 h, and were then treated with insulin (100 nM) for 30 min. Note that the pAkt bands are less intense in extracts of serum starved cells and enhanced in the extracts of insulin stimulation after serum starvation. Actin was used as a loading control. The amounts of pFoxO1 ^T24^ relative to total FoxO1 protein and the amounts of pAkt^S473^ relative to total Akt protein were calculated relative to those in the cell extracts from control siRNA-transfected cells.

As the present data suggest that Mdm20 regulates mTORC2 through its effect on Rictor expression, we investigated whether FoxO1 is also regulated by the Mdm20-mTORC2-Akt pathway. FoxO1 has multiple phosphorylation sites and is phosphorylated by several proteins, including Akt, Protein kinase A (PKA), SGK, cyclin-dependent kinase 2 (CDK2), and creatine kinase (CK) [31–34]. Among these kinases, Akt phosphorylates FoxO1 at Thr24, Ser256, and Ser319, and suppresses FoxO1 nuclear transportation [35, 36]. Thus, we examined the cellular localization of FoxO1 in Mdm20-KD cells (Fig 7A). In the control cells, FoxO1 was more predominantly localized to the nucleus than to the cytoplasm (Fig 7A-c). By contrast, it was mostly localized to the cytoplasm in both Mdm20- and Nat5-KD cells (Fig 7A-f and 7A-i). Conversely, it was localized to the nucleus in Rictor-KD cells, similar to the localization observed in control cells (Fig 7A-l). We next examined pFoxO1 levels using an anti-phosho-FoxO1 antibody (Fig 7B). To compare pFoxO1 levels among siRNA-transfected cells, we measured the amounts of pFoxO1 and FoxO1, and calculated the ratios of pFoxO1/FoxO1 in Mdm20-KD, Nat5-KD, and Rictor-KD cells. Consistent with the subcellular localization of FoxO1, pFoxO1^T24^ levels in both Mdm20- and Nat5-KD cells were 1.5- and 1.3-fold higher, respectively, than that in control cells, whereas these levels were lower in Rictor-KD cells, even though the pAkt ^S473^ level was decreased in both Mdm20- and Rictor-KD cells.

Akt activation is regulated by the IGF-PI3K signaling pathway in response to serum starvation or insulin stimulation. The IGF-PI3K signaling pathway is down-regulated under serum-starvation conditions, but is up-regulated by insulin stimulation. We examined the relationship between Akt activation and FoxO1 phosphorylation under serum starvation and insulin stimulation conditions using siRNA oligonucleotide-transfected cells. Upon serum starvation, the pAkt^S473^ level in control and Nat5-KD cells, was reduced to less than 25% of that observed under control conditions, but its level increased to 85% after insulin stimulation. However, upon of serum starvation of Mdm20-KD cells, the level was less than 5% of that observed under control conditions and only recovered to 20% in response to insulin stimulation. Similar to pAkt^S473^, pFoxO1^T24^ levels in Mdm20-KD cells under serum starvation and insulin stimulation conditions were 15% and 88%, respectively, of those under control conditions, and were markedly lower than those found in control cells (46% and 135%, respectively). In the case of Rictor-KD cells, pFoxO1^T24^ levels were lower than those found in control cells under normal culture (71%), serum starvation (13%) and insulin stimulation conditions (62%). These data suggest that Mdm20 affects FoxO1 activity through the mTORC2-Akt pathway in response to nutrient stimulation.

## Discussion

**Fig 8:**
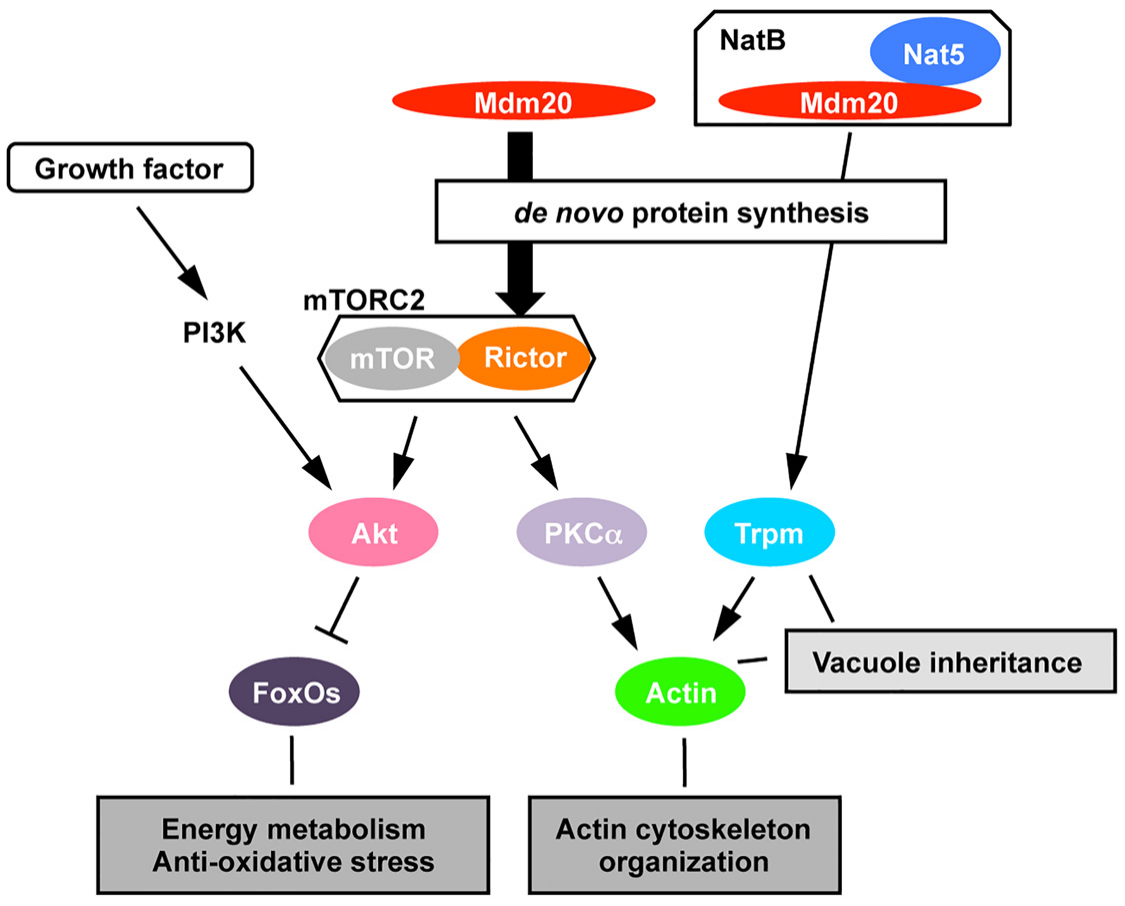
Novel regulation of actin remodeling by Mdm20 through the mTORC2 pathway. Mdm20 (magenta) is required for actin (green) cable stabilization through the N-acetylation of Trpm (light blue), which is mediated by NaB activity and involved in vacuole inheritance through actin-Trpm fibers. However, the present data show that Mdm20 deficiency suppresses mTORC2 activity by reducing Rictor (orange) expression, which may be regulated during *de novo* protein synthesis by the Nat5 (blue)-independent pathway. Because mTORC2 activates PKCα (light purple) and regulates actin (green) organization and cellular motility, these data indicate that Mdm20 modulates actin remodeling and cellular motility through the mTORC2 pathway. Additionally, Mdm20 regulates not only pAkt (pink), but also pFoxO1 (dark purple), through its effects on mTORC2 activity in response to serum starvation and insulin stimulation. Our findings show that Mdm20 acts as novel regulator of mTORC2 via its ability to regulate Rictor expression and is independent of NatB.

The most interesting finding of our study is that Mdm20 modulates mTORC2 activity via its ability to affect Rictor expression. Our data show for the first time that Mdm20 has novel biological functions in cellular homeostasis that are independent of those of Nat5, and that Mdm20 can function as an auxiliary NatB subunit. mTOR plays critical roles in cellular processes related to cell growth, metabolism, tumorigenesis, neurodegenerative diseases, cellular senescence, organismal aging and age-related diseases [7, 8]. mTOR exists in two functionally distinct protein complexes, mTORC1 and mTORC2. mTORC1 affects fundamental cellular functions, including protein synthesis, autophagy, glucose homeostasis, lipogenesis, and mitochondrial functions, after it has been activated by growth factors, amino acids, or changes in cellular energy status [37, 38]. By contrast, mTORC2 modulates cellular survival pathways and cellular motility through Akt and PKCα phosphorylation, respectively. However, unlike the mTORC1 signaling pathway, the upstream regulators of mTORC2 signaling are unknown. Rictor is a specific component of the mTORC2 complex. In Mdm20-KD cells, the Rictor protein level was markedly reduced and mTORC2 activity was suppressed, suggesting that Mdm20 plays a role in modulating mTORC2 activity. In fact, both pAkt and pPKCα levels were decreased in Mdm20-KD cells. Akt is also activated by the insulin/IGF-PI3K signaling pathway and is involved in cell survival, proliferation, glucose metabolism and cell migration via many other factors, including mTOR, FoxOs, p21 and GSK3β. We previously showed that Mdm20 suppresses protein aggregate clearance by inhibiting the induction of autophagy through Akt^S473^ phosphorylation [19]. In the present study, Mdm20 deficiency was shown to reduce FoxO1 phosphorylation by Akt in response to serum starvation and insulin stimulation (Fig 8). Although FoxO1 was localized to the cytoplasm, and pFoxO1 levels were higher in both Mdm20- and Nat5-KD cells in culture medium containing 10% FBS, FoxO1 is also phosphorylated by PKA, not Akt [32]. Caesar et al. reported that a *∆nat3* mutant strain has low PKA activity resulting from the inhibition of the N-terminal acetylation of Tfs1p, a stress induced carboxylpeptidase Y (CPY) inhibitor and phosphatidylethanolamine-binding protein in yeast [39].

Mdm20 is required as part of the NatB complex together with Nat5 for actin remodeling via the N-acetylation of Trpm. As shown in Fig 3, Mdm20 is required for Trpm fiber assembly. Furthermore, the number of vacuoles was higher in Mdm20- and Nat5-KD cells than in the control cells. The increase in the number of vacuoles was accompanied by the disappearance of Trpm assembly points near the perinuclear region and was closely associated with a deficiency in NatB components. In yeast, a similar phenotype is observed in *∆nat3*, *act1*, and *tpm1* mutant strains [16]. Actin is involved in directed vacuole movement during cell division via its interaction with myosin and Trpm [40]. Liu et al. also reported that Trpm is directly involved in vesicular transport [41, 42]. Taken together, these data suggest that the increased formation of vacuoles in Mdm20- and Nat5-KD cells is caused by Trpm assembly dysfunction via the suppression of N-terminal acetylation by NatB. However, vacuole formation did not correlate with stress fiber formation or the suppression of cellular motility in Mdm20-KD cells. These findings indicate that Mdm20 modulates actin remodeling and cellular motility independent of Trpm disassembly by NatB. PKCα is a regulator of actin cytoskeleton organization and is activated by mTORC2 [29, 30]. The pPKCα^S657^ level was reduced in Mdm20-KD cells, but not in Nat5-KD cells, suggesting that Mdm20 may also play a role in regulating cellular motility via actin cytoskeleton organization by the mTORC2-PKCα pathway (Fig 8).

We found that Mdm20 deficiency reduces Rictor protein levels. In addition, the Nat5 protein level was also reduced in Mdm20-KD cells. The lower amounts of Rictor and Nat5 protein were not recovered by treating the Mdm20-KD cells with protease inhibitors. By contrast, Rictor and Nat5 mRNA expression in Mdm20-KD cells were similar to those of control cells. Taken together, these data suggest that the regulation of Rictor and Nat5 expression by Mdm20 may not be linked to protein degradation or changes in transcriptional regulation, but to *de novo* protein synthesis. Although the mechanism by which Mdm20 modulates the translation of Rictor and Nat5 is unknown, one possibility is that Mdm20 interacts with catalytic subunits of the Nat family other than Nat5, resulting in a complex that functions to regulate Rictor and Nat5 translation.

The maintenance of protein, energy, and metabolic homeostasis, as well as protective mechanisms against various environmental stresses, such as oxidative stress, is important for counteracting the effects of aging. The dysregulation of these biological processes is closely linked to age-related diseases, such as neurodegenerative diseases, diabetes and cancer. Akt and mTOR are key regulatory kinases in the aging process because they have a wide variety of substrates that mediate numerous biological functions. The most important finding in this study is the identification of Mdm20 as a novel regulator of mTORC2. Understanding the role of Mdm20 in this pathway will provide a new direction for uncovering the molecular basis of anti-aging and long lifespan, whose mechanisms may not be the same as those functioning in age-related diseases.

## Materials and Methods

### Cell culture and transfection

HEK293, HeLa, HeLa stably expressing Fucci and HepG2 cells (from JCRB Cell Bank, Japan) were maintained at 37°C in a 5% CO_2_ humidified atmosphere and were grown in Dulbecco’s modified Eagle’s medium (DMEM) containing 10% fetal bovine serum. Transfection was performed using Lipofectamine and Plus reagents (Invitrogen) at densities of 5 **×** 10^4^ cells and 1.5 **×** 10^5^ cells per well in 24- and 6-well plates, respectively. All of the cell lines were cultured for 72 h after transfection. To perform siRNA transfection, a 20 mM solution of each oligonucleotide was added to the transfection cocktail (25 nM, final conc.) and transfected into the cells using Lipofectamine 2000 (Invitrogen). Following the transfection, the cells were cultured for 72 h.

### siRNA oligonucleotides

siRNA oligonucleotides targeting Mdm20, Nat5 and Rictor were chemically synthesized (Integrated DNA Technologies/IDT), and the sequences are shown in S1 Table. Control siRNA was purchased from IDT.

### Antibodies and chemicals

Rabbit polyclonal antibody of Mdm20 was generated against the C-terminal peptide of human Mdm20 protein, which is conserved between human and mouse, fused with GST protein. The antiserum was purified using GST and protein A sepharose, columns, as described previously [20]. Anti-Nat5 (human homologue of yeast Nat3) (65145) antibody was purchased from Santa Cruz. The other antibodies used in this study were purchased as follows: anti-β-actin (A5441) antibody was purchased from Sigma; anti-Trpm (74480) and anti-phospho-PKCα (12356) antibodies were purchased from Santa Cruz; anti-phospho-Akt (Ser473 (4060), and T308 (2965)), anti-Akt (4691), anti-PKCα (2056), anti-mTOR (2983), anti-Rictor (2114), anti-Raptor (2280), anti-LST8/GβL (3274), anti-FoxO1 (2880), anti-phospho-FoxO1 (2599), anti-Cofilin (5175), and anti-phospho-Cofilin (3313) antibodies were purchased from Cell Signaling Technology; anti-LC3 (L7543) antibody was purchased from Medical and Biological Laboratories (MBL); anti-ubiquitin (MAB1510) antibody was purchased from Millipore; and anti-paxillin (610052) antibody was purchased from BD Transduction Laboratory. MG132 was purchased from Sigma, and ammonium chloride was purchased from Nacalai Tesque.

### Immunoblotting

Following the chemical treatments, cells were washed with PBS and lysed with RIPA buffer containing 50 mM Tris-Cl (pH 7.5), 150 mM NaCl, 5 mM EDTA, 1% NP-40, 0.1% SDS, 1 mM DTT, phosphatase inhibitor mix (Nacalai Tesque) and protease inhibitor cocktail (Nacalai Tesque) on ice. The cell lysates were subjected to immunoblotting with the indicated antibodies, and the immune complexes were detected using a chemiluminescence reagent (GE Healthcare).

### Immunofluorescence analysis

HEK293 or HeLa cells were fixed with 4% paraformaldehyde and then permeabilized by treatment with PBS and 0.2% Triton-X100. The cells were blocked with 1% albumin in PBS and then incubated with primary antibodies. Subsequently, the cells were treated with one of the following appropriate secondary antibodies: Alexa 488 donkey anti-rabbit IgG, Alexa 594 donkey anti-rabbit IgG, Alexa 594 donkey anti-mouse IgG, Alexa Fluor 488 phalloidin, or Alexa Fluor 568 phalloidin (Molecular Probes). Cellular DNA was counterstained with DAPI (Dojindo). Images were captured using an Axioskop2 plus (Zeiss) fluorescent microscope equipped with Axiovision software.

### Cell cycle analysis using Fucci-expressing HeLa cells

Fucci-expressing HeLa cells were transfected with siRNA oligonucleotides and incubated for 72 h. At 24 h intervals, the transfected cells were fixed with 4% paraformaldehyde and analyzed using an Axioskop2 plus (Zeiss) fluorescent microscope equipped with Axiovision software. The S/G2/M and G1 phases were observed using Alexa Fluor 489 and Alexa Fluor 546 filters, respectively. Merged images of the cells from both filters were used to analyze the G1/S interphase.

### Cell migration assay

Seventy-two hours after transfecting HeLa and HepG2 cells with the control or two types of Mdm20 siRNA oligonucleotides, KD cells were harvested and suspended in serum free medium. The cell suspension (2.5 **×** 10^5^ cells) was placed in the upper chamber, and 10% FBS/DMEM was applied to the lower chamber using Transwell polycarbonate membrane (pore size: 8 μm) cell culture inserts (Corning). Cells were incubated for 6 h and then fixed with 3.7% formaldehyde. After the removal of non-migratory cells, the migratory cells were stained with 0.4% Crystal violet/10% ethanol and quantified.

### Quantitative real-time PCR analysis

Total RNA was extracted from cultured cells using an RNeasy Mini Kit (Qiagen) according to the manufacturer’s instructions. One microgram of total RNA was reverse transcribed using a Transcriptor First Strand cDNA Synthesis Kit (Roche). Quantitative PCR was performed using LightCycler480 SYBR Green I Master (Roche) with gene specific primer sets purchased from TaKaRa Bio. Amplification and real-time detection were performed using a LightCycler480 System (Roche). The expression of the target genes was normalized to that of human glyceraldehyde-3-phosphate dehydrogenase (GAPDH), which was used as an endogenous control.

### Statistical analysis

Statistical analyses were performed using Microsoft Excel. Comparisons were made using the t-test. The data were presented as the mean ± standard deviation (S.D.). *P* values of less than 0.01 were considered to be statistically significant.

## Supporting Information

S1 Fig Effect of NatB deficiency on actin cytoskeleton organization. Immunohistochemistry shows the cellular localization of actin (upper panels: a-c) and mRFP (middle panels: d-f) following co-transfection of mRFP and siRNA oligonucleotides into HeLa cells. The cells were fixed, and the images were captured 72 h post-transfection. Merged images of cells counterstained with DAPI (blue) are shown in panels g, h, and i. The arrows indicate the stress fibers. (Scale bar: 20 μm.)
(TIF)

S2 Fig Cofilin localization in Mdm20-KD HeLa cells. The distribution of actin and cofilin in Mdm20-, Nat5-, and Rictor-KD HeLa cells is shown. After fixing HeLa cells with paraformaldehyde, the cells were immunostained with phalloidin (green) and anti-Cofilin (red) antibodies. Merged images of cells counterstained with DAPI (blue) are shown in panels g, h, and i. (Scale bar: 20 μm.)
(TIF)

S1 Table The sequences of the siRNA oligonucleotides. Numbers indicate the position from the translational start of each mRNA.
(DOC)
