# Supplementary figures and images for: Mdm20 Modulates Actin Remodeling through the mTORC2 Pathway via Its Effect on Rictor Expression
Source: PLoS One. 2015 Nov 23;10(11):e0142943. doi: 10.1371/journal.pone.0142943 (PMC4658088; doi:10.1371/journal.pone.0142943)

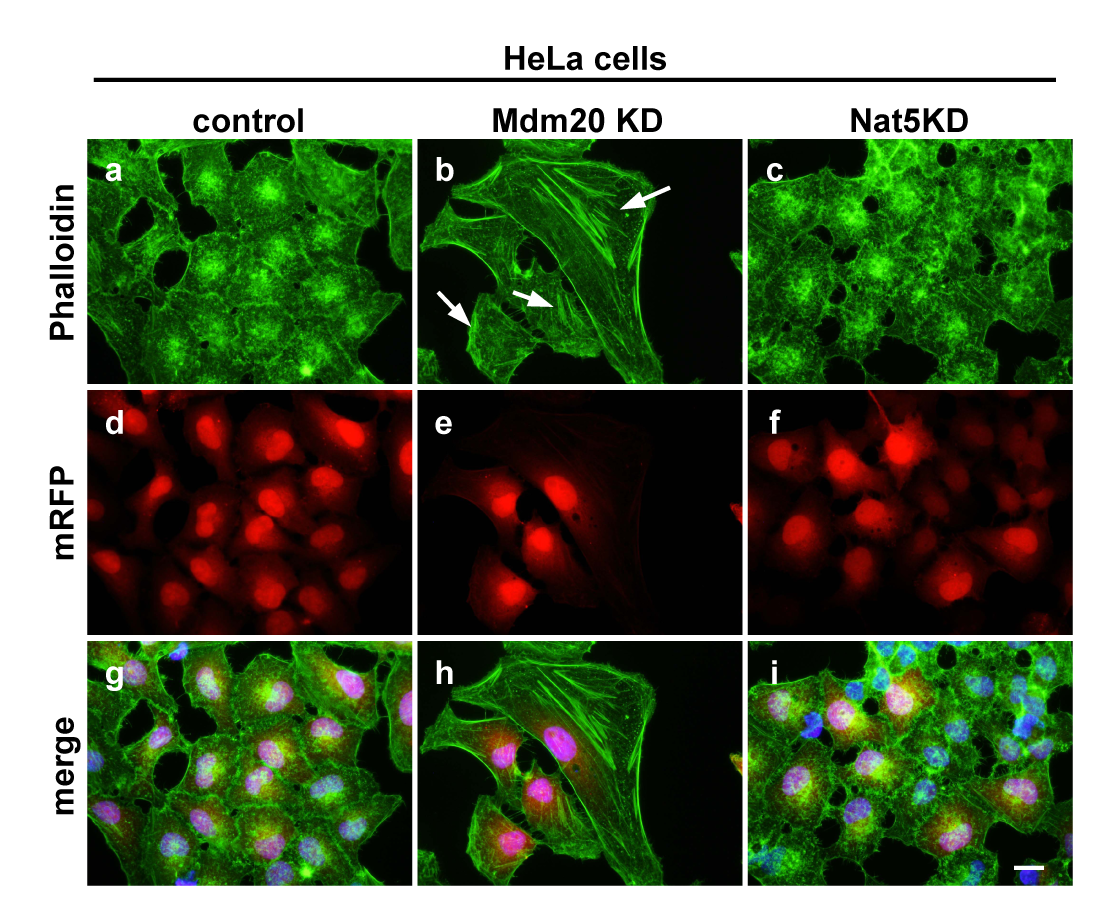

Supplement: S1 Fig — Immunohistochemistry shows the cellular localization of actin (upper panels: a-c) and mRFP (middle panels: d-f) following co-transfection of mRFP and siRNA oligonucleotides into HeLa cells. The cells were fixed, and the images were captured 72 h post-transfection. Merged images of cells counterstained with DAPI (blue) are shown in panels g, h, and i. The arrows indicate the stress fibers. (Scale bar: 20 μm.) (TIF) [file pone.0142943.s001.tif]

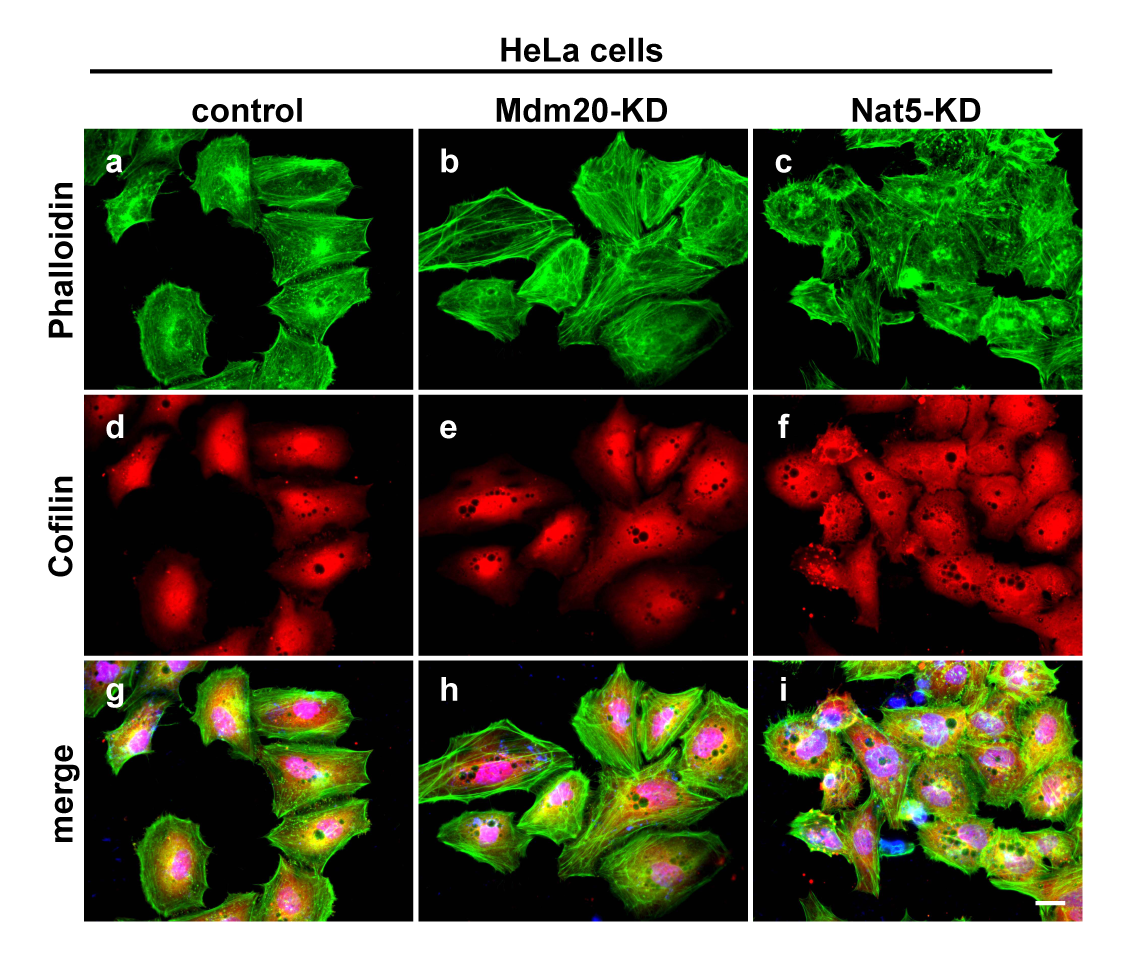

Supplement: S2 Fig — The distribution of actin and cofilin in Mdm20-, Nat5-, and Rictor-KD HeLa cells is shown. After fixing HeLa cells with paraformaldehyde, the cells were immunostained with phalloidin (green) and anti-Cofilin (red) antibodies. Merged images of cells counterstained with DAPI (blue) are shown in panels g, h, and i. (Scale bar: 20 μm.) (TIF) [file pone.0142943.s002.tif]
