# Supplementary material for: Mdm20 Modulates Actin Remodeling through the mTORC2 Pathway via Its Effect on Rictor Expression
Source: PLoS One. 2015 Nov 23;10(11):e0142943. doi: 10.1371/journal.pone.0142943 (PMC4658088; doi:10.1371/journal.pone.0142943)
Supplement: S1 Table — Numbers indicate the position from the translational start of each mRNA. (DOC) [file pone.0142943.s003.doc]

| Mdm20-KD1 (1774-1792) | 5’-AGCGAUAAACUCUGGGAUC-3’ |  |  |
| --- | --- | --- | --- |
| Mdm20-KD2 (329-347) | 5’-UGAUACUCCUCACUAUUGG-3’ |  |  |
| Nat5-KD1 (67-85) | 5’-CUUACAGAAACUUAUGGGA-3’ |  |  |
| Nat5-KD2 (45-63) | 5’-CAACAACAUUAACUUGGAU-3’ |  |  |
| Rictor-KD (5055-5073) | 5’-GCAUGAAGAAGCAGAGGCU-3’ |  |  |
